# Isolation by Elevation: Genetic Structure at Neutral and Putatively Non-Neutral Loci in a Dominant Tree of Subtropical Forests, *Castanopsis eyrei*

**DOI:** 10.1371/journal.pone.0021302

**Published:** 2011-06-20

**Authors:** Miao-Miao Shi, Stefan G. Michalski, Xiao-Yong Chen, Walter Durka

**Affiliations:** 1 Helmholtz Centre for Environmental Research – UFZ, Department of Community Ecology (BZF), Halle, Germany; 2 School of Resources and Environmental Sciences, Tiantong National Station for Forest Ecosystem, East China Normal University, Shanghai, China; University of Melbourne, Australia

## Abstract

**Background:**

The distribution of genetic diversity among plant populations growing along elevational gradients can be affected by neutral as well as selective processes. Molecular markers used to study these patterns usually target neutral processes only, but may also be affected by selection. In this study, the effects of elevation and successional stage on genetic diversity of a dominant tree species were investigated controlling for neutrality of the microsatellite loci used.

**Methodology/Principal Findings:**

Diversity and differentiation among 24 populations of *Castanopsis eyrei* from different elevations (251–920 m) and successional stages were analysed by eight microsatellite loci. We found that one of the loci (Ccu97H18) strongly deviated from a neutral model of differentiation among populations due to either divergent selection or hitchhiking with an unknown selected locus. The analysis showed that *C. eyrei* populations had a high level of genetic diversity within populations (*A_R_* = 7.6, *H_E_* = 0.82). Genetic variation increased with elevation for both the putatively selected locus Ccu97H18 and the neutral loci. At locus Ccu97H18 one allele was dominant at low elevations, which was replaced at higher elevations by an increasing number of other alleles. The level of genetic differentiation at neutral loci was similar to that of other Fagaceae species (*F_ST_* = 0.032, 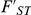 = 0.15). Population differentiation followed a model of isolation by distance but additionally, strongly significant isolation by elevation was found, both for neutral loci and the putatively selected locus.

**Conclusions/Significance:**

The results indicate higher gene flow among similar elevational levels than across different elevational levels and suggest a selective influence of elevation on the distribution of genetic diversity in *C. eyrei*. The study underlines the importance to check the selective neutrality of marker loci in analyses of population structure.

## Introduction

Genetic composition within and among populations is shaped by the interplay of genetic drift, gene flow, mutation and natural selection. Molecular markers have helped to identify the effect of life history traits, phylogeographic history and environmental factors on the genetic structure of plant populations [1], [2]. Among environmental factors, abiotic factors, such as soil type, topology or elevation, play an important role in genetic structuring because they may affect phenology, population size or density and thus gene flow or genetic drift [3]. Elevation is of particular importance, and many studies focused on its relationships with plant performance and phenotype [4], but also on genetic variation of molecular markers [3], [5], [6].

Genetic variation within populations often varies along elevational gradients and among species different patterns have been identified [7]. First, mid-elevation populations may hold higher levels of diversity compared with both low and high elevation populations due to the optimal mid-elevation habitats following the central-marginal hypothesis (e.g. [8]). Second, low elevation populations may have highest diversity which decreases with elevation as a result of bottlenecks occurring throughout upward range expansion (e.g. [9]). Third, highest genetic diversity was found at high elevations which was attributed to various reasons like decreased human disturbance and/or historical downward range shifts due to climate change, and adaptation [5], [7]. Lastly, genetic variation also has been found to stay rather constant along a given elevational gradient due to extensive gene flow (e.g. [10]). Overall, these inconsistent patterns support a predominant role of life history traits and of biogeographic history in determining patterns of genetic variation along elevational gradients. The processes underlying these patterns are either neutral, like genetic drift and bottleneck effects as a result of the demographic history, or are selective due to the climatic clines related to elevation.

Elevational clines encompass a suite of environmental factors that are either physically linked with elevation like temperature [11] or that are instead correlated with it, like land use [4]. Depending on the ability of these factors to exert selection or to affect the neutral processes of gene flow and drift, molecular markers may display elevational patterns. Of the various molecular markers, which have been used to study genetic variation, microsatellites are assumed to represent neutral markers because microsatellites are generally found in non-coding regions [12] and are characterized by high levels of variability. Consequently, patterns of differentiation among populations at microsatellite loci are almost exclusively interpreted as genetic drift and gene flow. However, some empirical studies indicated the presence of non-neutral microsatellite loci [12], [13], [14]. Thus, in order to study neutral processes the neutrality of loci should be confirmed before performing other genetic analyses [7], [15]. Due to the steep clines in environmental conditions with increasing elevation accompanied by changes in selective conditions, non-neutral behaviour of individual molecular markers is likely, e.g. due to physical linkage to specific genes under selection (e.g. [16]).

In mixed and evergreen broad-leaved subtropical forests of Southeast Asia, *Castanopsis eyrei* (Fagaceae) is often the dominant tree species in late successional forests. The long lived evergreen species is native to southeastern China and Taiwan and occurs along a large elevational gradient from <300 m to 1700 m a.s.l. (http://www.efloras.org/florataxon.aspx?flora_id=620&taxon_id=200006236). It is monoecious and wind-pollinated and the acorn seeds are predominantly dispersed by gravity and small rodents [17]. Due to these life history traits, *C. eyrei* populations are expected to have high genetic diversity and efficient gene flow mediated by pollen dispersal should result in low levels of genetic differentiation.

In this study we examined the distribution of genetic variation in *C. eyrei* populations within a nature reserve of continuous mixed broad-leaved forest across a mountain range. Specifically, we ask (1) whether individual loci are more strongly differentiated than expected from a neutral model, and (2) whether spatial structure, elevation or successional stage affect the patterns of neutral and of putatively adaptive genetic variation, respectively.

## Results

### Identification of loci under selection

**Figure 1:**
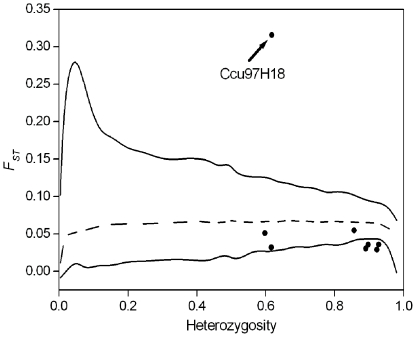
*F_ST_* values of eight microsatellite loci in *Castanopsis eyrei* populations plotted against heterozygosity. The lines represent the median (broken line) and 99% quantiles of expected *F_ST_* values estimated from a neutral model [15].

Outlier tests performed using FDIST detected a significant departure of the *F_ST_* value from neutral expectations for locus Ccu97H18 (*F_ST_* = 0.316, Fig. 1), while for other loci *F_ST_* values ranged from *F_ST_* = 0.029 to 0.055. However, four of them with lower *F_ST_* values were also situated out of the simulated distribution, which was probably due to the extremely high value of Ccu97H18. When we excluded this locus and reanalysed the other seven loci, the result confirmed their neutrality as all of them were situated within the 0.99 quantile.

**Figure 2:**
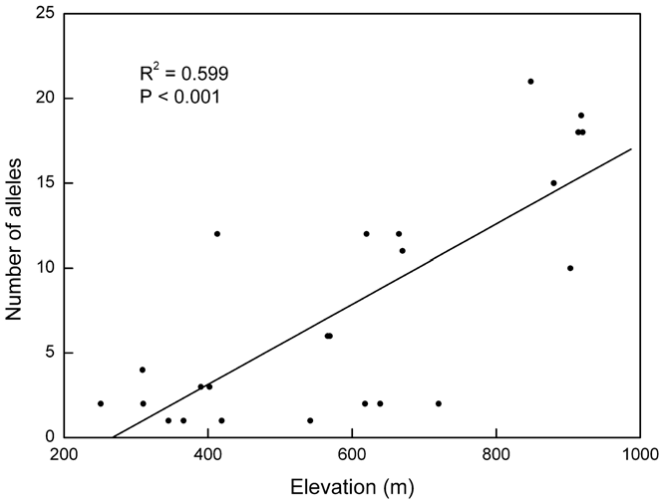
Number of alleles per population of *Castanopsis eyrei* as a function of elevation at microsatellite locus Ccu97H18.

Analysing only locus Ccu97H18, we found an increase in the number of alleles with elevation from an average of 2.2±1.2 below 400 m a.s.l. to 16.8±3.9 above 800 m a.s.l (Fig. 2). This was due to one allele in particular (145 bp) which was most common with frequency close to 1.0 at lower elevations (< ca. 700 m), whereas its frequency decreased drastically at higher elevations.

### Genetic diversity at species and population level

Genetic parameters at species and population levels for both the putatively neutral loci and the putatively selected locus Ccu97H18 are displayed in Table 1. In a total of 583 individuals and at the seven putatively neutral loci, we identified 129 alleles with a number of 10 to 25 alleles per locus. At the population level, the mean number of alleles per locus ranged from 6.1 to 12.1 (mean  = 9.4) and allelic richness (*A_R_*) varied from 5.4 to 7.7 (mean  = 6.7). The expected heterozygosity (*H_E_*) ranged from 0.68 to 0.86 among populations (mean  = 0.78). At the species level, *C. eyrei* had a *H_E_* value of 0.82. The bottleneck analyses indicated recent reduction in population size in five sites (Table 1), which were located at low, medium and high elevations.

**Table 1:** Sample sites and genetic diversity estimates for *Castanopsis eyrei* samples.

					Neutral loci				Ccu97H18		
Population	location	Elevation (m)	Successional stage	*N*	*A*	*A_R_*	*H_E_*	*H_Eeq_*	*A*	*A_R_*	*H_E_*
CSP 02	E 118.13484, N 29.24926	390	5	20	10.1	7.2	0.78	0.656	3	2.3	0.11
CSP 03	E 118.12402, N 29.23885	720	3	18	9.1	6.8	0.76	0.852	2	1.8	0.06
CSP 04	E 118.12015, N 29.24963	542	5	22	10.0	7.3	0.86	0.004**	1	1.0	0.00
CSP 06	E 118.14747, N 29.25497	880	3	49	12.7	7.4	0.81	0.406	15	8.0	0.75
CSP 07	E 118.14373, N 29.25184	903	4	21	10.0	7.1	0.81	0.656	10	9.3	0.91
CSP 08	E 118.11019, N 29.24106	413	3	17	8.4	6.5	0.78	0.055	12	10.9	0.91
CSP 10	E 118.15791, N 29.25188	670	4	18	8.9	6.7	0.79	0.289	11	9.7	0.90
CSP 12	E 118.12190, N 29.24939	620	4	18	8.4	6.5	0.78	0.344	12	10.7	0.90
CSP 13	E 118.11621, N 29.24630	402	5	34	10.6	6.3	0.74	0.852	3	1.9	0.09
CSP 14	E 118.13518, N 29.24944	639	5	24	9.0	6.4	0.78	0.289	2	1.8	0.10
CSP 15	E 118.13106, N 29.24917	618	5	17	6.1	5.4	0.69	0.188	2	2.0	0.08
CSP 16	E 118.09966, N 29.24253	309	1	15	8.1	6.8	0.78	0.531	4	3.6	0.21
CSP 17	E 118.10828, N 29.24342	310	2	17	6.6	5.7	0.76	0.012*	2	1.7	0.06
CSP 18	E 118.12461. N 29.24516	569	3	18	7.3	6.1	0.79	0.039*	6	4.6	0.31
CSP 21	E 118.08084, N 29.27059	566	5	45	10.7	6.5	0.77	0.289	6	3.1	0.20
CSP 23	E 118.13723, N 29.21450	419	2	19	8.0	6.2	0.77	0.594	1	1.0	0.00
CSP 24	E 118.13469, N 29.21483	366	1	20	8.1	6.3	0.78	0.469	1	1.0	0.00
CSP 25	E 118.13155, N 29.21713	345	2	12	6.1	5.4	0.68	0.813	1	1.0	0.00
CSP 26	E 118.12155, N 29.21489	251	1	17	8.7	6.7	0.79	0.711	2	1.7	0.06
CSP 27	E 118.13605, N 29.24709	665	5	47	12.1	6.9	0.79	0.813	12	7.4	0.76
A	E 118.14136, N 29.24811	848	5	28	11.0	7.6	0.85	0.004**	21	14.8	0.96
B	E 118.14381, N 29.25244	914	5	27	11.9	7.4	0.83	0.406	18	13.1	0.94
C	E 118.14345, N 29.25249	918	5	30	11.4	7.5	0.83	0.148	19	14.1	0.96
D	E 118.14306, N 29.25265	920	5	30	11.9	7.7	0.85	0.004**	18	13.2	0.94
Mean				24	9.4	6.7	0.78		7.7	5.8	0.43
All				583	18.4	7.6	0.82		29	8.6	0.43

N, number of individuals sampled per population; A, mean number of alleles; A_R_, allelic richness; H_E_, expected heterozygosity;. H_Eeq_, expected heterozygosity under drift-migration equilibrium and TPM with significant departure indicated by asterisks. *P<0.05, **P<0.01.

### Effects of environmental factors

Successional stage was significantly interrelated with elevation (*r* = 0.567, P = 0.004 Spearman correlation). Over all neutral loci, the multiple regression of allelic richness (*A_R_*) against elevation and successional stage showed that *A_R_* increased significantly with elevation but succession had no significant contribution (*r* = 0.586, P = 0.005). For the putatively selected locus Ccu97H18, similarly only elevation had a significantly positive strong effect on *A_R_* in the mutliple regression analysis (*r* = 0.708, P<0.001).

### Population differentiation

**Figure 3:**
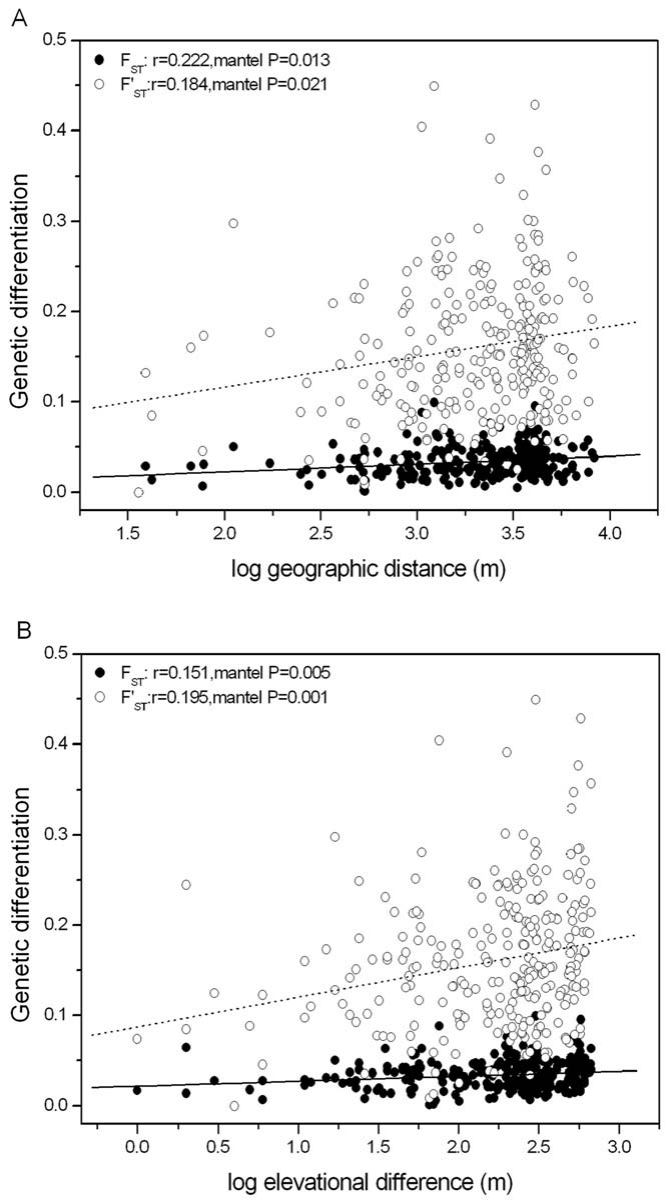
Patterns of isolation by distance and isolation by elevation in *Castanopsis eyrei*. Population differentiation (closed circle and solid line: pairwise *F_ST_*; open circle and dotted line: standardized 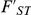) at neutral loci as a function of (A) geographic distance and (B) as a function of elevational difference. Correlation coefficient and Mantel-*P*.

Populations were significantly structured as revealed by overall *F_ST_* over the seven neutral loci of 0.032 (P<0.01). However, the standardized 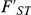 value was 0.15, indicating considerable differentiation. When only the putatively selected locus Ccu97H18 was analysed, we detected much higher values of *F_ST_* = 0.316 and 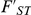 = 0.571. Significant patterns of isolation by geographic distance were found at neutral loci both for pairwise *F_ST_* and 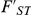
values (Fig. 3). For the putatively selected locus Ccu97H18, a significant pattern of isolation by distance was detected for pairwise *F_ST_* (*r* = 0.193, P = 0.016), but the pattern did not exist for pairwise 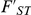 (*r* = 0.069, P = 0.194). When we checked for a pattern of isolation by elevational distance, both pairwise *F_ST_* and pairwise 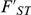 revealed much more strongly significant correlations, indicating isolation by elevation, both in the neutral loci (Fig. 3) and the putatively selected locus (*F_ST_*: *r* = 0.251, P = 0.002; 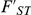: *r* = 0.210, P = 0.003). Since elevational distance was correlated with geographic distance (*r* = 0.329, P = 0.002), we performed partial Mantel tests to test whether elevational distance was related to genetic differentiation after accounting for geographic distance. For the neutral loci, elevational distance remained significant for pairwise 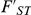
(*r* = 0.129, P = 0.010) but not for pairwise *F_ST_* (*r* = 0.060, P = 0.123). For the putatively selected locus elevational distance remained significantly related to differentiation after accounting for geographic distance in both *F_ST_* and 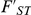
(*F_ST_*: *r* = 0.188, P = 0.013; 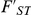: *r* = 0.201, P = 0.001).

## Discussion

### Neutrality of microsatellite loci

Microsatellites are assumed to represent ideal neutral markers, so that only gene flow and genetic drift rather than selection should affect their genetic structure. However, an increasing number of studies indicated the presence of non-neutral loci [14], [18], [19]. In the present study one out of eight loci that were originally developed for *C. cuspidata* var. *sieboldii* [20], [21] showed non-neutral behaviour. However, no information on the genomic position and putatively linked genes of this locus is available (Ueno pers. comm.). Based on the analysis of expressed genes of *C. sieboldii*, Ueno et al. [22] showed that microsatellites are widespread with 314 microsatellites in 2417 potential unigenes. Consequently, microsatellite markers may be linked to expressed genes and, hence, tests of neutrality should precede population genetic analyses. Since only a limited number of microsatellite loci are routinely analysed in such studies and given that average linkage disequilibrium is expected to be low in outcrossing species, the likelihood of finding a marker linked to an adaptively important gene may be low [16]. However, based on studies that used the method of Beaumont and Nichols [15] to identify non-neutral microsatellite loci in plants, between 4% (one out of twenty six for *Fucus serratus* [23]) and 33% (three out of nine for *Astronium urundeuva* [24]) of loci were found to behave non-neutrally. However, these seemingly high levels of non neutral loci may be overestimated as the identification of outlier loci with non-neutral behaviour also produces false-positives [25], which should be controlled e.g. by correlating allele frequencies with potentially selective site conditions (e.g. [26]).

### Genetic diversity of *Castanopsis eyrei*

At the seven neutral microsatellite loci employed in this study a total of 129 alleles were detected with 10 to 25 alleles found per locus. Ueno et al. [20], [21] detected a total of 78 alleles in *C. cuspidata* with these same loci in a limited number of individuals. In our study, *C. eyrei* showed many more alleles than *C. cuspidata* in the original work, possibly due to the larger sample size (*N* = 583 and *N* = 24 for *C. eyrei* and *C. cuspidata*, respectively).

Genetic variation at the species level in *C. eyrei* was high (*H_E_* = 0.82) and similar to that of other congeneric species like *C. cuspidata* (*H_E_* = 0.83 [27]), and *C. acuminatissima* (*H_E_* = 0.72 [28]). These species share common characteristics like an outcrossing mating system, wind pollination and a long life span. Furthermore, they are all climax species with a broad current distribution and thus may also have similar demographic histories. Species exhibiting these traits are generally expected to show high levels of genetic variation [1].

### Population structure

Focusing on neutral genetic variation and thus, excluding the putatively selected locus, overall population differentiation was low (*F_ST_* = 0.032) indicating only little differentiation [29]. However, the adjusted 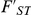 [30] equaled 0.15 for the neutral loci. Such values would indicate substantial differentiation, quite unexpected for perennial, wind pollinated and outcrossing species. However, other tree species of the Fagaceae that were analysed with microsatellite markers show similar values with a mean of 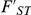 = 0.145 (Table 2). Levels of differentiation derived from dominant markers are somewhat lower (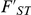 = 0.124 for AFLP or RAPD markers, Table 2) and drastically lower when estimated from allozyme markers (mean 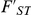 = 0.054, Table 2). This discrepancy indicates that the absolute level of 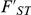 values has to be interpreted with caution, e.g. marker specific mutation rates have to be taken into account. In fact it seems unlikely that across the scale of a few kilometres populations of these tree species are strongly differentiated in neutral markers because of extensive pollen flow and seed dispersal by animals.

**Table 2:** Genetic differentiation among populations of tree species in the family Fagaceae as assessed with different molecular markers.

species	*N*	*H_S_*	*F_ST_*	*F_ST max_*	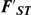	Reference
*microsatellites*						
*Castanea crenata*	6	0.780	0.034	0.190	0.179	[58]
*Castanopsis acuminatissima*	3	0.716	0.006	0.209	0.029	[28]
*Cyclobalanopsis myrsinaefolia*	5	0.553	0.061	0.393	0.155	[59]
*Fagus crenata*	23	0.839	0.027	0.155	0.174	[47]
*Fagus japonica*	16	0.659	0.023	0.327	0.070	[60]
*Lithocarpus densiflorus*	19	0.540	0.090	0.447	0.202	[61]
*Quercus garryana*	22	0.596	0.063	0.393	0.160	[62]
*Quercus glauca*	10	0.741	0.042	0.239	0.176	[63]
*Quercus robur*	7	0.868	0.018	0.115	0.156	[64]
*allozymes*						
*Cyclobalanopsis championii*	5	0.151	0.092	0.818	0.112	[65]
*Cyclobalanopsis glauca*	6	0.222	0.065	0.745	0.087	[66]
*Fagus crenata*	23	0.187	0.038	0.806	0.047	[67]
*Fagus sylvatica*	38	0.250	0.030	0.745	0.040	[68]
*Quercus acutissima*	3	0.152	0.010	0.788	0.013	[69]
*Quercus chrysolepis*	6	0.443	0.018	0.511	0.035	[70]
*Quercus petraea*	21	0.381	0.027	0.607	0.044	[71]
*AFLP/RAPD*						
*Castanopsis fargesii*	5	0.283	0.043	0.670	0.064	[72]
*Cyclobalanopsis glauca*	4	0.315	0.104	0.620	0.167	[73]
*Lithocarpus harlandii*	3	0.232	0.227	0.688	0.330	[74]
*Quercus petraea*	4	0.289	0.021	0.649	0.032	[75]
*Quercus petraea*	21	0.233	0.024	0.758	0.032	[71]
*Quercus robur*	4	0.284	0.021	0.654	0.033	[75]
*Quercus robur*	7	0.190	0.110	0.785	0.140	[64]
*Trigonobalanus verticillata*	3	0.153	0.153	0.787	0.194	[76]
**mean SSR**		**0.699**	**0.040**	**0.274**	**0.145**	
**mean allozyme**		**0.255**	**0.040**	**0.717**	**0.054**	
**mean AFLP/RAPD**		**0.247**	**0.088**	**0.701**	**0.124**	

The absolute levels of standardized pairwise population differentiation, 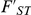, approached unity in several cases at the putatively selected locus Ccu97H18. This demonstrates that these population pairs are almost fixed for different alleles, a fact that is not obvious with traditional *F_ST_*. However, the relationship between population differentiation and spatial or elevational distance was almost the same for the traditional and standardized *F_ST_* values. Thus a more comprehensive understanding of differentiation patterns is possible using standardized differentiation measures [31], [32].

### Isolation by elevation

Significant isolation by distance was found for the neutral loci and locus Ccu97H18 (only for pairwise *F_ST_*). However, additionally significant isolation by elevation was detected in both the potentially adaptive locus and the non-adaptive loci after accounting for the effect geographic distance. This pattern of isolation by elevation suggests higher rates of gene flow among sites at similar elevations than along elevational clines [3]. Elevation can result in reproductive isolation due to phenological shifts, e.g. delayed budding [33] or shift of flowering time or prolonged floral longevity and stigma receptivity [34] resulting in temporal separation of the timing of flowering [35]. Phenological differences in flowering time in turn will lead to partial reproductive isolation which both may facilitate adaptation to elevation and lead to neutral genetic differentiation as has been shown for other forest trees [36].

Populations of *C. eyrei* at the top of the mountains harboured the largest amount of genetic variation whereas populations at lower elevation had reduced levels of variation. Although not often observed among trees [7] a similar pattern was found in other tree species [16], [37], [38]. As both the non-selected loci and the putatively selected locus displayed the same pattern, a number of non-mutually exclusive processes may have contributed. First, mutation rate may be higher at higher elevations due to increased ultraviolet-B radiation [7]. If effective, this process should apply to all loci in a similar manner and may have contributed to the general trend across all loci. However, microsatellites are polymorphic due to slippage mutation of the DNA polymerase and UV radiation seems not necessarily to affect this process [39], [40]. Second, human disturbance is much stronger at lower elevations. Charcoal has been detected in many local soil profiles [41] indicating past fire clearance. Populations at higher elevations are more rarely influenced by human activities and, thus, are able to preserve genetic diversity. We found a significant positive correlation between elevation and successional stage, indicating that older, less disturbed forests are often located in higher elevations. Hence, it is likely that undisturbed upland forests served as sources for colonization after logging at low elevations. Recent and older bottleneck and founder effects may thus have contributed to reduced variation at lower elevations. However, bottleneck tests did not support the hypothesis of recent reductions of population size at lower elevation. However, in wind pollinated trees, large gamete pools may be involved in colonization, maintaining high levels of diversity in colonized areas (e.g. [42]). Third, selection may be a significant force. Locus Ccu97H18 showed a strong cline as the most common allele at low elevations almost went extinct at higher elevations and many other alleles appeared instead. These patterns are unlikely due to random genetic drift or restricted gene flow, but most likely due to selection. Since Ccu97H18 is a short microsatellite, genetic hitchhiking is the most probable reason for the observed patterns, assuming that the locus is linked with loci under selection, as has been shown for other microsatellite loci in trees [43], [44], [45]. We do not have evidence on the genes potentially involved. Thus, both the target of selection and the potential contribution of diversifying selection producing the cline and/or balancing selection bearing high allelic diversity remain obscure. Overall, the study suggests that elevation can be an efficient driver for genetic differentiation at both neutral and adaptive loci at the landscape scale.

## Materials and Methods

### Ethics Statement

Field work and the collecion of leaves were performed in cooperation with and under approval by the Gutianshan National Nature Reserve in China.

### Study area and populations

Our study was carried out in Gutianshan National Nature Reserve (NNR) located in the western part of Zhejiang Province, China (29°8′–29°17′ N, 118°2′–118°11′ E). *C. eyrei* is the dominant tree species in the area and continuously distributed throughout [46]. The Gutianshan NNR has an area of approximately 81 km^2^ with elevations ranging from 250 to 1250 m a.s.l.. It mainly consists of species-rich evergreen broad-leaved forests including old growth forest and successional stages that developed after cease of human use in 1975 [41]. In 2008, we sampled 24 representative sites of 30×30 m which were spread across all successional stages and the local elevational range of the species (251–920 m). We did not sample at >1000 m a.s.l. because the species was too rare. Five successional stages were distinguished according to the average age of the tree layer ([41], 1: <20 yrs, 2: <40 yrs, 3: <60 yrs, 4: <80 yrs, 5: ≥ 80 yrs). Additional details of site selection and conditions for 20 of the sites are reported in Bruelheide et al. [41]. We sampled all mature individuals of *C. eyrei* inside the sites and some additional individuals outside of sites CSP 6 and CSP 21 because there were too few inside. In each of the 24 populations, 12 to 49 individuals (mean  = 24) were collected, totalling 583 individuals (Table 1).

### Population genetic analysis

Total genomic DNA was isolated from ca. 10 mg dried leaf material using the DNeasy 96 plant extraction kit (QIAGEN) following manufacturers instructions. Samples were genotyped at eight microsatellite loci previously developed for *C. cuspidata* var. *sieboldii* [20], [21]. Multiplex polymerase chain reactions (PCR) were performed in a total volume of 10 µl. Ccu16H15 (Label: PET, redesigned reverse primer: GAAATTGAGTTGGGTTAGTTCCAC), Ccu28H18 (FAM), Ccu62F15 (NED), Ccu33H25 (FAM), Ccu90T17 (PET), Ccu102F36 (VIC) and Ccu87F23 (FAM) were in one PCR amplification. Another single PCR amplification was performed for Ccu97H18 (VIC). Thermocycler protocol was one cycle of 95°C for 15 min, followed by 35 cycles of 30 s at 94°C, 90 s at 58°C and 1 min at 72°C, with a final extension of 20 min at 72°C. PCR products from the two amplifications were mixed and separated on an ABI 3130 genetic analyzer (Applied Biosystems) with internal size standard GeneScan™ 600 LIZ. Individuals were genotyped using GeneMapper version 3.7 (Applied Biosystems). *C. eyrei* is diploid and none of the samples displayed more than two alleles.

Because the study species is a wind pollinated perennial tree of the Fagaceae, many of which exhibit populations in Hardy-Weinberg equilibrium (HWE, e.g. [47], [48]), we assumed that *C. eyrei* microsatellite loci should conform to HWE. Because trans-species amplification of microsatellites often results in null alleles we checked the data for the presence of null alleles under the assumption of HWE using MICRO-CHECKER [49]. Except for Ccu16H15, all loci showed signs of null-alleles, the frequency of which ranged from 1.3% to 20% (mean  = 6.99%). We took three approaches to deal with null-alleles. First, we adjusted homozygous single locus genotypes, if necessary, by adding an additional allele in the frequency of the null-allele. This approach assumes that there is one single null allele, which is treated as an additional allele. Second, we used the null allele correction procedure of the FreeNA software [50] to calculate pairwise *F_ST_* values. This approach corrects for null alleles but restricts the analysis to the visible alleles. Third, we left data unchanged. However, all subsequent analyses showed similar results irrespective of the type of null allele treatment. Therefore, we only present the results of the MICRO-CHECKER approach as it allows the calculation of standard diversity descriptors.

We tested the eight microsatellite loci for outliers, i.e. markers potentially under selection, using the program FDIST [15]. A null distribution of target *F_ST_* values expected from a neutral model is generated and quantile limits are calculated. Loci outside a 99% confidence interval are regarded as potentially under selection. Following Acheré *et al*. [18], the neutral expectation was first based on the observed overall mean *F_ST_* calculated from all markers. In a second step, the overall mean *F_ST_* was recalculated without the putatively selected locus and used as target value for a new null distribution to test the remaining loci. As our analyses suggested that locus Ccu97H18 was potentially under directional selection, we performed all the following analyses both for the seven loci conforming to a neutral model (“neutral loci”) and only for locus Ccu97H18.

Genetic diversity at population level was characterized by number of alleles (*A*), allelic richness (*A_R_*, correcting for sample size by rarefaction for a minimum sample size of 12) and expected heterozygosity (*H_E_*) using FSTAT version 2.9.3.2 [51]. Because genotypes were adjusted for null-alleles, we did not calculate inbreeding coefficients. In the dataset of neutral loci we tested for recent bottlenecks (reductions of effective population size) by testing for an excess of heterozygosity relative to expectations of a mutation–drift equilibrium [52]. We used the software BOTTLENECK [53] and applied the recommended two-phase mutation model (TPM) with 70% stepwise and 30% multistep mutations, a variance of 12, 1000 iterations in the coalescent simulations and one-tailed Wilcoxon's signed-rank tests. To assess population differentiation, pairwise *F_ST_* values based on Weir and Cockerham's [54] estimator *θ* were calculated using FSTAT. As *F_ST_* is likely to underestimate genetic differentiation between populations for markers which show high levels of allelic variability, we calculated 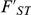, a standardized parameter of genetic differentiation as 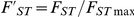 [30]. 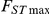 was calculated after recoding the data using RECODEDATA [55]. To test for isolation by distance [56], the association between pairwise genetic differentiation (*F_ST_*) and pairwise geographic distances (log transformed) was tested using the Mantel test implemented in R 2.8.1 [57]. We also performed a Mantel test between *F_ST_* and pairwise elevational differences (log transformed) to test for isolation by elevational distance. Since pairwise elevational difference was correlated to pairwise geographic distance, we performed partial Mantel tests to test for effects of elevation after accounting for geographic distance. Because allelic diversity differed between populations and was correlated with elevation, this may have biased the estimates of pairwise differentiation using *F_ST_*. We therefore calculated standardized pairwise *F_ST_* values (pairwise 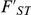, [30], eqn. 4b) and repeated the tests for isolation by distance and isolation by elevation. In order to compare the genetic differentiation of *C. eyrei* with other species of the Fagaceae, we reviewed empirical studies and calculated 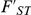 following Hedrick ([30], eqn. 4b).

### Statistical analysis

To test the effects of environmental factors on genetic variation, we analysed the relationship between allelic richness (*A_R_*) and the two predictors elevation and successional stage in a multiple regression. We used *A_R_*, which corrects for sample size, rather than *H_E_*, because sample size varied among populations; however, the results were qualitatively the same for *H_E_*. Collinearity of elevation and successional stage was checked by Spearman correlation. All analyses were performed with R 2.8.1 [57].
